# Fusing Events and Frames with Coordinate Attention Gated Recurrent Unit for Monocular Depth Estimation

**DOI:** 10.3390/s24237752

**Published:** 2024-12-04

**Authors:** Huimei Duan, Chenggang Guo, Yuan Ou

**Affiliations:** School of Computer and Software Engineering, Xihua University, Chengdu 610039, China; 212022083500001@stu.xhu.edu.cn (H.D.);

**Keywords:** monocular depth estimation, event cameras, coordinate attention, gate recurrent units

## Abstract

Monocular depth estimation is a central problem in computer vision and robot vision, aiming at obtaining the depth information of a scene from a single image. In some extreme environments such as dynamics or drastic lighting changes, monocular depth estimation methods based on conventional cameras often perform poorly. Event cameras are able to capture brightness changes asynchronously but are not able to acquire color and absolute brightness information. Thus, it is an ideal choice to make full use of the complementary advantages of event cameras and conventional cameras. However, how to effectively fuse event data and frames to improve the accuracy and robustness of monocular depth estimation remains an urgent problem. To overcome these challenges, a novel Coordinate Attention Gated Recurrent Unit (CAGRU) is proposed in this paper. Unlike the conventional ConvGRUs, our CAGRU abandons the conventional practice of using convolutional layers for all the gates and innovatively designs the coordinate attention as an attention gate and combines it with the convolutional gate. Coordinate attention explicitly models inter-channel dependencies and coordinate information in space. The coordinate attention gate in conjunction with the convolutional gate enable the network to model feature information spatially, temporally, and internally across channels. Based on this, the CAGRU can enhance the information density of the sparse events in the spatial domain in the recursive process of temporal information, thereby achieving more effective feature screening and fusion. It can effectively integrate feature information from event cameras and standard cameras, further improving the accuracy and robustness of monocular depth estimation. The experimental results show that the method proposed in this paper achieves significant performance improvements on different public datasets.

## 1. Introduction

Monocular depth estimation [1,2,3,4] is a critical and challenging task in computer vision, which is a crucial aspect in applications such as robot navigation [5,6,7], autonomous driving [8], and 3D reconstruction [9]. Nowadays, frame-based monocular depth estimation performs well in everyday scenes [10,11]. However, in high-speed motion scenes and some extreme environments, it is difficult to obtain high-quality image information with standard cameras that suffer from motion blur and high exposure, which makes it difficult for the traditional monocular depth estimation methods to handle these challenging scenes. Event cameras [12], such as bio-inspired vision sensors, work by capturing the intensity changes at the pixel level and returning them as an asynchronous stream of events. Compared to standard cameras, event cameras only work at the pixel level and do not need to wait for global exposure, so they have the advantages of high dynamic range, high temporal resolution, and no motion blur. Therefore, several event-based methods have been proposed for monocular depth estimation [13,14,15]. Although these methods show superiority in some extreme environments, event cameras are more favorable for extracting features at texture regions and boundaries. It is not possible to obtain the global context of the scene in static environments. Therefore, predicting depth from events alone still has some limitations. On the contrary, standard cameras are good at global information acquisition. Combining the complementary characteristics of conventional and event cameras and fusing the two types of image information in deep learning are expected to improve the accuracy of monocular depth prediction while solving multiple challenges such as occlusion, motion blur, and exposure.

To take advantage of the complementary nature of events and frames, recent work has attempted to fuse events and frames for monocular depth estimation [16,17,18]. In terms of the fusing methods, EVEN [17] targets poor lighting conditions such as nighttime by performing the pixel-by-pixel addition of low-light-enhanced nighttime RGB images and event images to obtain a fused image and then predicting the depth from the fused image. However, pixel-by-pixel addition does not take into account the spatial and temporal contextual information between modalities, especially at the edges of the image or in regions with complex textures, and may not accurately preserve the fine structure in the original image. SRFNet [18] further adopts a spatial-reliability-based fusion approach. The method acquires spatial priors for frames and events separately and then learns the fused features interactively through an attention layer. However, this interactive learning model focuses on the local details of each modality. Considering that frames and event data are asynchronous and irregular, RAMNet [16] employs a ConvGRU [19] to maintain the asynchronous updating of the state of the frames and events, thus cleverly fusing the information from the events and frames. With their unique gating mechanism, GRUs exhibit excellent ability to capture long-term dependencies in sequential data, making them ideal for handling such complex asynchronous data fusion tasks. However, in the case of the ConvGRU [19] employed in RAMNet, its reset gate and update gate are implemented by a convolutional layer, which neglects the modeling of inter-channel relationships. Moreover, limited by the local receptive field of convolution, the ConvGRU still cannot effectively capture global contextual information.

In order to solve the above problems, this paper proposes a novel Coordinate Attention Gated Recurrent Unit (CAGRU). In this unit, we design an attention gate using coordinate attention [20]. Coordinate attention can explicitly model inter-channel dependencies and extract global spatial context information by encoding horizontal and vertical coordinate information. Using it as a gate enables the network to focus on the key regions in the image as information flows. Our CAGRU integrates the attention gate into the ConvGRU and retains one of the original convolutional gates in the ConvGRU, enabling the attention gate and convolutional gate to cooperate in controlling the flow of information to keep the state of events and frames updated. In this way, the CAGRU is able to model features in temporal, spatial, and inter-channel aspects, which enables more accurate and efficient feature filtering during information transfer and further effectively fuses the feature information from events and frames. Our attention gate can be used as either an update gate or reset gate in the CAGRU, and thus we have two variants of CAGRU: CAGRU-U and CAGRU-R. We embed the CAGRU into a dual-stream feature extraction network framework based on UNet [16]. The framework processes event streams and video frames independently through encoders specific to different sensors. Then, the CAGRU is used to update and fuse the features and states obtained from the encoders. Finally, the fused features are fed to the decoders to obtain the prediction depth. We report the performance of our method on two datasets, EventScape [16] and DENSE [14]. The experimental results show that our monocular depth estimation method using a CAGRU to fuse frames and events greatly outperforms the RAMNet method using a ConvGRU on both datasets and outperforms other state-of-the-art event and frame fusion approaches based on monocular depth estimation. The contributions of this paper are as follows:

- A Coordinate Attention Gated Recurrent Unit (CAGRU) is proposed. By integrating coordinate attention into a ConvGRU and replacing the convolutional gate with the coordinate attention gate, two variants of CAGRUs are formed. The effectiveness of the coordinate attention mechanism in improving the performance of GRU models is demonstrated.
- A monocular depth estimation network fusing events and frames with a CAGRU is implemented, which can explicitly model channel internal relationships and global context information, effectively fusing information from events and frames. The superiority and robustness of our network are verified on two benchmark datasets.

## 2. Related Work

### 2.1. Frame-Based Monocular Depth Estimation

The early monocular depth estimation studies shifted from cue-based (e.g., vanishing point [21], focus and defocus [22], and SFS [23], etc.) to machine-learning-based (e.g., CRF [24], MRF [25], and SIFT [26], etc.) but still have limitations due to their reliance on external assumptions. Monocular depth estimation research has entered into a new phase after the introduction of deep learning. Initially, complex CNNs (e.g., VGG [27], ResNet [28,29,30], and DenseNet [31,32]) were used for monocular depth estimation. In order to make full use of temporal information, there are many works using recurrent network architectures to extract spatio-temporal features from images [33,34,35]. They add ConvLSTM or LSTM layers to the encoder and alternate with convolutional layers to jointly extract spatio-temporal features. There are also approaches [36,37,38] that incorporate a recurrent unit between the CNN encoder and the decoder, which updates the features from the encoder, and the decoder recovers the depth with the updated features. With the context modeling capability of the self-attention mechanism, transformers have also been introduced into the field of monocular depth estimation [39,40,41]. Although monocular depth estimation methods based on deep learning have achieved good performance, they still have difficulties under unfavorable conditions such as strong light and high speed due to the inherent drawbacks of traditional RGB cameras.

### 2.2. Event-Based Monocular Depth Estimation

Monocular depth estimation has become increasingly important in recent years with advances in fields such as robot navigation [12,42,43] and autonomous driving [44], where the need for low-latency obstacle avoidance and high-speed route planning has increased, especially using event cameras. The development of event-based monocular depth estimation research is very similar to that of frame-based, starting from early multi-view stereo [45] or model-based methods [46,47], which require the use of auxiliary information to obtain sparse or semi-dense depth maps. Later, deep-learning-based methods were proposed to obtain dense depth maps, from using CNNs to predict depth from asynchronous events [14,48,49,50] to the introduction of recurrent network architectures to establish temporal contextual clues [14,51] and then to the introduction of transformers to realize the interaction of spatio-temporal information [35,52,53]. These learning methods select appropriate event representations (e.g., tensor voxel meshes) so that they can be processed by deep learning frameworks. However, data acquisition based only on RGB frames or events may still limit performance gains. In addition, from an architectural design perspective, CNNs cannot fully exploit the rich temporal information of events; RNNs have weak encoding capabilities for spatial information, and transformer has high computational costs regarding high-resolution prediction tasks.

### 2.3. Fusion of Events and Frames

In recent years, fusing events and frames has been widely explored in a variety of visual tasks, such as depth estimation [16,17,18,54,55,56,57], feature tracking [58,59,60,61], visual odometry [59,62], and image deblurring [63]. Most of these methods are based on a two-stream-encoder feature extraction network, where specific encoders are designed for event data and frame data, respectively, to extract intermediate features, which are then fused. The advantage of this is that it enables the targeted processing of the data of different modalities, which is conducive to feature extraction. However, events are asynchronous while frames are synchronized, and the frequency of both occurrences is random. A suitable fusion approach is required to preserve the characteristics of the two modalities, which is still a challenging problem.

In terms of the fusion methods, some fuse two modality features through simple mathematical operations, such as FE-Fusion-VPR [64] using a concatenation operation and EVEN [17] using pixel-level summation. Others incorporate an attention mechanism. For example, Bimodel SegNet [65] uses a cross-attention mechanism, Ref. [66] uses a self-attention mechanism, and SRFNet [18] guides the fusion of modalities by using the spatial priors of the events and frames as an initial mask and uses the attention layer to learn the consensus in the mask. RAMNet [16] maintains an asynchronously updating hidden state via a ConvGRU, which enables the fusion of frame and event information, and has yielded significant results in monocular depth estimation. For asynchronous and irregular information like events and frames from multiple sensors, a recurrent unit that enables the asynchronous updating of state information is a good option. However, traditional GRUs use the same convolution operation for all gates, ignoring inter-channel relationship modeling and limiting the global context modeling capability. We therefore propose a CAGRU using coordinate attention. The CAGRU designs the coordinate attention as a gate to model the inter-channel relationships of features, and this gate is coupled with a convolutional gate to extract global contextual information. We asynchronously update the hidden state of the events and frames through the CAGRU to achieve information fusion.

### 2.4. Gated Recurrent Units

RNNs can memorize temporal context information at different time steps. However, in many computer vision tasks, the sequences to be processed are very long, and the use of traditional RNNs is prone to gradient vanishing or explosion problems. To solve this problem, researchers have introduced different gate mechanisms into RNNs, enabling the network to control the path of information transfer when processing sequence data. The earliest gated recurrent network was Long Short-Term Memory (LSTM) [67], which consisted of three gates (forget gate, input gate, and output gate) and a cell state. Since then, most gated recurrent units have innovated in terms of the number and use of gates [68,69,70,71,72], including GRU [73], which is also a simplified version of LSTM. A GRU achieves efficient and effective updates of hidden states using only a reset gate and update gate. In order to achieve spatial coding in image processing tasks, the ConvLSTM [74] and ConvGRU [19] were proposed by replacing the fully connected operations with convolutional operations in the recurrent unit. In recent years, a variety of methods have been proposed to improve the gating mechanism and candidate hidden state generation in the ConvGRU update formula [52,75,76,77,78]. GRViT [52] introduces a linear attention mechanism during cell state updating. Compared with the above methods that improve the number of gates, usage, and candidate state generation, we consider a new gate generation method from a simpler and more effective perspective.

## 3. Methods

**Symbol description:** In the following sections, ${\hat{I}}_{k}$ denotes the event image and *I* denotes the grayscale image. *f* denotes the encoded features, and *h* denotes the hidden state. The subscripts *e* and *i* of *f* and *h* denote the event image and the grayscale image, respectively. *k* denotes the time step of the input sequence. The superscript *j* denotes the resolution scale of the feature. In Section 3.3, *A*, *U*, and *R* denote coordinate attention gates, update gates, and reset gates, respectively, and the subscript *k* denotes the recurrent time step. $\tilde{h}$ is the candidate state. In the process of coordinate attention encoding, *z* is used to denote the features, and the superscripts *h* and *w* of *z* denote the features in vertical and horizontal directions, respectively.

### 3.1. Event Representation

Event cameras, such as DVS [79] and DAVIS [80], are bio-inspired visual sensors. When the brightness change at pixel point (x,y) at moment *t* exceeds a threshold *C*, the event camera reports an event e=(x,y,t,p) in the form of a quaternion. *p* is the polarity of the event *e* with a value of 1 or −1 to indicate the direction of the brightness change. After a time period, these events form an event stream S. To be able to process the event stream using our network, we need to transform it into a sequence of event images $\left\{{\hat{I}}_{k}\right\}$, where ${\hat{I}}_{k}\in {\left[0,1\right]}^{W\times H}$. To achieve this, we first divide the event stream S into non-overlapping event windows $\left\{S_{k}\right\}$ at a fixed time interval and then convert each event window $S_{k}$ into an event image ${\hat{I}}_{k}$ [81]. We discretize the duration ΔT of an event window into *B* event temporal bins so that each event image is a tensor of size H × W × B. We set B=5.

### 3.2. Overall Network Architecture

**Figure 1:**
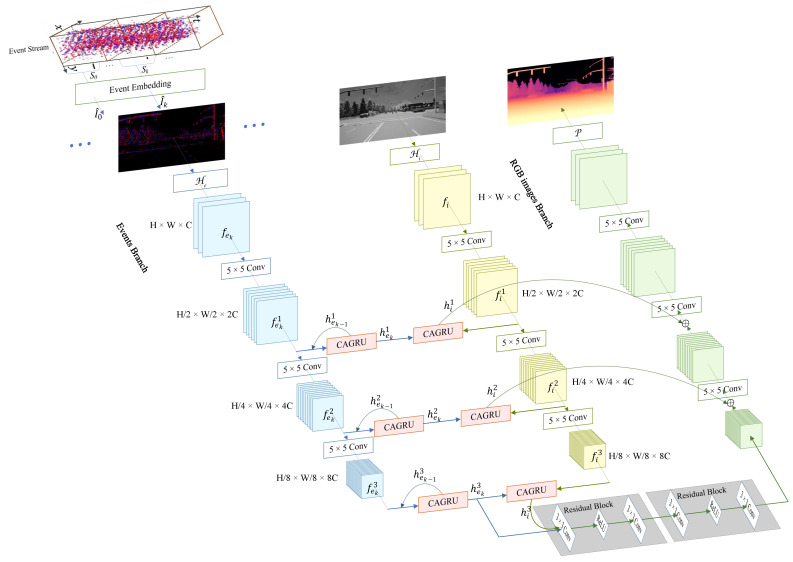
Overall network framework. Our network is a two-stream-input U-shaped encoder–decoder network. The event image branch, the frame image branch, and the decoding branch are denoted in blue, yellow, and green, respectively.

The working schematic of our network is shown in Figure 1. Since the sensors that provide event images and frames are different and measured at different rates, we use a specific encoder for each input to obtain intermediate features. Each input sequence consists of five event images and one grayscale image.

Specifically, an input sequence is denoted as $I=\{{\hat{I}}_{0},{\hat{I}}_{1},{\hat{I}}_{2},{\hat{I}}_{3},{\hat{I}}_{4},I\}$, where ${\hat{I}}_{k}\in R^{H\times W\times 5}$ and $I\in R^{H\times W\times 1}$. We first transform the event image ${\hat{I}}_{k}$ and the grayscale image *I* into initial features $f_{e_{k}}\in R^{H\times W\times 32}$ and $f_{i}\in R^{H\times W\times 32}$ through a header layer H (a 5×5 convolutional layer). Then, three 5×5 convolutional layers are used to encode the intermediate features at three scales, respectively.

Next, for the intermediate features of events and frames, we use the sensor-specific state fusion operator, CAGRU, to update the state to ensure that features from different sensors are mapped to the appropriate space and can be efficiently fused with the previous hidden state.

Specifically, the intermediate feature of the event image ${\hat{I}}_{k}$ is represented as $f_{e_{k}}^{j}\in R^{\frac{H}{2^{j}}\times \frac{W}{2^{j}}\times 2^{j}C}$, where j∈[1,3],k∈[0,4] and *j* denotes the resolution scale. At each scale, the state fusion unit CAGRU is used to iterate through these intermediate features in a loop to obtain the fused state. Taking the *j*th scale as an example, the intermediate feature $f_{e_{k}}^{j}$ of the event image ${\hat{I}}_{k}$ and the state $h_{e_{k-1}}^{j}$ of the previous event image ${\hat{I}}_{k-1}$ are jointly sent into the CAGRU to obtain the updated state $h_{e_{k}}^{j}$ of ${\hat{I}}_{k}$:

(1) $$h_{e_{k}}^{j}=\mathrm{CAGRU}\left(f_{e_{k}}^{j},h_{e_{k-1}}^{j}\right)$$

The intermediate feature of the grayscale image *I* is represented as $f_{i}^{j}\in R^{\frac{H}{2^{j}}\times \frac{W}{2^{j}}\times 2^{j}C}$, where j∈[1,3]. Taking the *j*th scale as an example, the state $h_{e_{4}}^{j}$ of the last event image ${\hat{I}}_{4}$ in the sequence acts as the previous state of the grayscale map *I* to update the state of *I*, which provides the fused state $h_{i}^{j}$:

(2) $$h_{i}^{j}=\mathrm{CAGRU}\left(f_{i}^{j},h_{e_{4}}^{j}\right)$$

**Figure 2:**
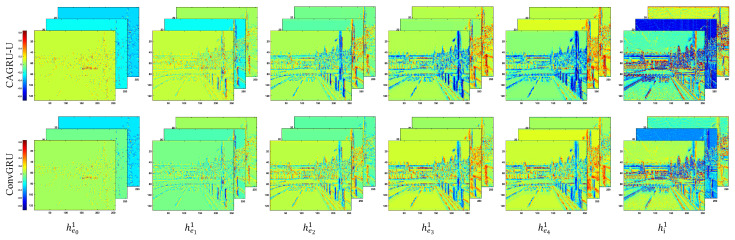
Comparison of hidden state changes when a sequence image passes through CAGRU-U and ConvGRU. (Hidden states are selected on the first scale and for the first three channels).

Through the recursive mechanism of CAGRU, spatially sparse event stream realizes information interaction and fusion in the time domain by the global channel reweighting of coordinate attention and performs final state update with the frame image, thus achieving effective cross-modal fusion. Finally, the fused states are fed to the decoder through skip connections to predict the depth. In Figure 2, we visualize the hidden state changes of using CAGRU-U and ConvGRU. It can be seen that our CAGRU can acquire more detailed features of the scene.

### 3.3. State Fusion Unit

Among most deep-learning-based monocular depth estimation methods, CNN-based methods have been highly successful in extracting features in the spatial dimension as convolution can efficiently enable spatial modeling between pixels. However, for the fusion of events and frames, the difference in data modality between the two makes it much more difficult to effectively extract features and predict depth. RAMNet [16] chooses ConvGRU to combine events and frames at the spatio-temporal level. However, a limitation of standard convolution is that it is inherently difficult to explicitly model channel relationships, while inter-channel relationships are crucial for extracting global features. We therefore integrate coordinate attention gates into ConvGRU, which together with conventional convolution gate forms our Coordinate Attention Gated Recurrent Unit (CAGRU). This unit is able to access channel information while spatio-temporally establishing long-term dependencies and enhances the feature representation of key areas by explicitly retaining coordinate information, thus improving the quality of features.

#### 3.3.1. Coordinate Attention Gate

Coordinate attention [20] is an attention mechanism that embeds location information into channel attention. It captures long-range dependencies and retains precise location information through a one-dimensional feature encoding process. Structurally, coordinate attention can be divided into two steps: coordinate information embedding and attention generation. Unlike the traditional channel attention mechanism that uses a 2D global pooling operation, coordinate attention employs two 1D average pooling operations to aggregate the input features into direction-aware feature maps along horizontal and vertical directions, respectively. Then, the two direction-aware feature maps are encoded as two separate attention maps, each capturing the distant dependencies of the input feature maps along one spatial direction. Thus, the location information is preserved in the generated attention maps. The two attention maps are then applied as weights to the input feature maps by multiplication to emphasize the representation of the attention regions. Therefore, in the state fusion unit, we make the weighted feature map go through a sigmoid activation function, which is designed as a gate to control the flow of information, called coordinate attention gate:

(3) A=σ(CA(X))

where CA denotes coordinate attention and σ denotes the sigmoid function.

**Figure 3:**
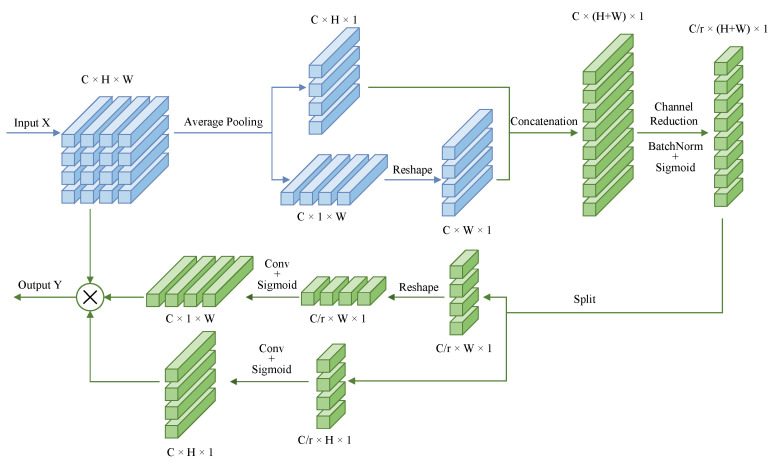
Schematic diagram of coordinate attention.

The feature encoding process for the coordinate attention gate is shown in Figure 3. We compress the given input *X* into two 1D features, $z^{h}\in R^{C\times H\times 1}$ and $z^{w}\in R^{C\times 1\times W}$, along the horizontal and vertical directions, respectively, by means of two average pooling layers. For one-dimensional average pooling along the horizontal direction, it calculates the average value of each channel at each height position, so $z^{h}$ retains the ordinate information. Since the global pooling operation is performed in the width direction, $z^{h}$ is able to capture the long-distance dependence in the width direction. Similarly, the one-dimensional average pooling in the vertical direction allows $z^{w}$ to retain the abscissa information and capture the long-distance dependence in the height direction. In order to make full use of the captured positional information and accurately highlight the region of interest, coordinate attention concatenates the two aggregated feature vectors in the spatial dimension to obtain a fusion feature map $z\in R^{C/r\times (H+W)\times 1}$. *r* is the channel reduction factor, which is used to compress the number of channels and control the size of the feature block, thus reducing the computational overhead. Therefore, through the concatenation in the dimension and the conversion of the number of channels, the fusion feature *z* contains the information between different channels so as to indirectly capture the dependencies between channels. In the process of generating the attention map, the fusion feature *z* is then split in spatial dimension into two separate tensors, $z^{h}\in R^{C/r\times H\times 1}$ and $z^{w}\in R^{C/r\times 1\times W}$. A convolution layer is then used to process $z^{h}$ and $z^{w}$, respectively, making their number of channels the same as that of *X*, thus obtaining $g^{h}\in R^{C\times H\times 1}$ and $g^{w}\in R^{C\times 1\times W}$, which are the generated attention weights. Finally, the weights are applied to the input *X*, which can be expressed as

(4) $$Y=X⊙g^{h}⊙g^{w}$$

where ⊙ denotes the Hadamard product.

Finally, we use a sigmoid function to map the attention-weighted feature map Y to between 0 and 1, forming a “gating” signal that controls the flow of information. When the output of the sigmoid function is close to 1, the gate is “on”, allowing information to pass through; when the output is close to 0, the gate is ‘off’, preventing information from passing through.

#### 3.3.2. ConvGRU

ConvGRU [19] utilizes two gates to control the information flow within the cell, namely the reset gate and the update gate. The update gate is used to control the retention of old information, which helps the model to capture long-term dependencies; the reset gate is used to control the inflow of new information, which helps the model to capture short-term dependencies. These two gates enable ConvGRU to effectively update the hidden state based on the sequence information, thus providing a significant advantage in dealing with sequence problems. Equation (5) shows the formula. $U_{k}$ and $R_{k}$ are the update gate and reset gate, respectively. ${\tilde{h}}_{k}$ is the candidate state, and $h_{k}$ is the hidden state. * denotes the 2D convolution operation. *W* and *b* represent the corresponding convolution kernel weights and biases, respectively. ⊙ denotes the Hadamard product, [·,·] denotes the concatenation, and σ is the sigmoid activation function.

(5) $$\begin{array}{cc} U_{k} & =\sigma (W_{u}*\left[h_{k-1},f_{k}\right]+b_{u})\\ R_{k} & =\sigma (W_{r}*\left[h_{k-1},f_{k}\right]+b_{r})\\ {\tilde{h}}_{k} & =\tanh\left(W_{h}*\left[\left(R_{k}⊙h_{k-1}\right),f_{k}\right]+b_{h}\right)\\ h_{k} & =\left(1-U_{k}\right)⊙h_{k-1}+U_{k}⊙{\tilde{h}}_{k} \end{array}$$

ConvGRU has been widely used in deep learning networks [16,82,83,84] and has been shown to be a key component in convolutional recurrent networks. However, its gates are only implemented with convolution layers and the sigmoid activation function. Since convolution has limited ability in modeling global contextual information and lacks modeling of inter-channel relationships, our approach is to innovate its reset gate or update gate by designing a coordinate attention gate, thereby forming a new recurrent unit, in which the attention gate is coupled with the traditional convolutional gate. This allows the model to selectively focus on more important information and more effectively control the retention or forgetting of information.

#### 3.3.3. Coordinate Attention Gated Recurrent Unit (CAGRU)

**Figure 4:**
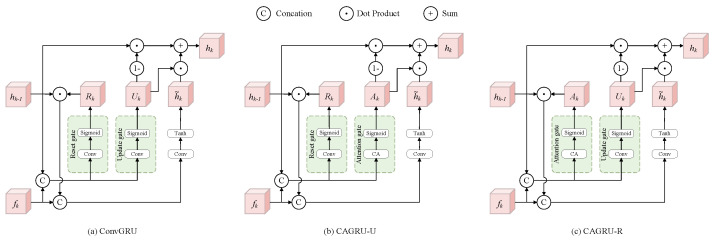
Comparison of the structures of the proposed CAGRU and ConvGRU.

Our CAGRU mainly consists of two core gates. On the one hand, the update gate or reset gate of ConvGRU (Figure 4a) is retained for establishing the connection between spatio-temporal domains. On the other hand, a new coordinate attention gate is designed for establishing the inter-channel relationship of the input features. Experiments have demonstrated that the coordinate attention gate can be used as either an update gate or a reset gate. Both work better than the original gate based on convolutional operations. Thus, we have two variants of CAGRU: CAGRU-U and CAGRU-R, as shown in Figure 4b,c.

In our network, CAGRU takes the *k*th feature $f_{k}$ and the previous hidden state $h_{k-1}$ as inputs and updates the current hidden state $h_{k}$, which can be expressed as

(6) $$h_{k}=\mathrm{CAGRU}\left(f_{k},h_{k-1}\right)$$

As shown in Figure 4b, CAGRU-U mainly consists of a reset gate and a coordinate attention gate. The reset gate is used to control the contribution of the previous state to the current candidate state and establish the short-range dependency between the two. The purpose of the attention gate is to control how much information is retained in the previous state and the current candidate state, respectively, and to establish long-range dependencies between the previous state and the current state. $h_{k-1}$ is first filtered by the reset gate $R_{k}$, then concatenated with $f_{k}$, and the candidate state ${\tilde{h}}_{k}$ is obtained through the convolution kernel and tanh function. The attention gate $A_{k}$ is then used to assign the weights of ${\tilde{h}}_{k}$ and $h_{k-1}$. It can be expressed as

(7) $$\begin{array}{cc} A_{k} & =\sigma (\mathrm{CA}\left[f_{k},h_{k-1}\right])\\ R_{k} & =\sigma (W_{r}*\left[h_{k-1},f_{k}\right]+b_{r})\\ {\tilde{h}}_{k} & =tanh(W_{h}*\left[\left(h_{k-1}⊙R_{k}\right),f_{k}\right]+b_{h})\\ h_{k} & =\left(1-A_{k}\right)⊙h_{k-1}+A_{k}⊙{\tilde{h}}_{k} \end{array}$$

As shown in Figure 4c, CAGRU-R mainly consists of a coordinate attention gate and an update gate. The attention gate controls the influence of the previous moment state on the current candidate state. The update gate controls how much the state information from the previous moment is retained to update the current hidden state and establishes long-range dependencies. Specifically, $h_{k-1}$ is first filtered by the attention gate $A_{k}$, then concatenated with $f_{k}$, and the candidate state ${\tilde{h}}_{k}$ is obtained through the convolution kernel and tanh function. The update gate $U_{k}$ is then used to assign the weights of ${\tilde{h}}_{k}$ and $h_{k-1}$, which can be formulated as

(8) $$\begin{array}{cc} A_{k} & =\sigma (\mathrm{CA}\left[f_{k},h_{k-1}\right])\\ U_{k} & =\sigma (W_{u}*\left[h_{k-1},f_{k}\right]+b_{u})\\ {\tilde{h}}_{k} & =tanh(W_{h}*\left[\left(h_{k-1}⊙A_{k}\right),f_{k}\right]+b_{h})\\ h_{k} & =\left(1-U_{k}\right)⊙h_{k-1}+U_{k}⊙{\tilde{h}}_{k} \end{array}$$

### 3.4. Loss Function

We train our network in a supervised manner. The labels are provided by the CARLA simulator while training on the EventScape and DENSE datasets. According to [16], we normalize the metric depth value $\hat{D}$ obtained from the network to obtain a pixel-level logarithmic depth mapping D∈[0,1] in order to facilitate the prediction of widely varying depths. The normalization can be expressed as

(9) $$D=\frac{1}{\alpha }\log\frac{\hat{D}}{D_{max}}+1$$

where α is a predefined parameter and $D_{max}$ denotes the maximum depth, which means that we only estimate the depth of the scene within the maximum depth range. We set α=5.7 and $D_{max}=1000$ following RAMNet [16].

We use scale invariance loss [85] and multi-scale scale-invariant gradient matching loss [11] to guide model training. As the monocular depth estimation task is inherently ill-posed, the use of scale-invariant loss maintains the consistency and stability of the output, and multi-scale scale-invariant gradient matching loss promotes the smoothing of edge variations. For pixel location *i*, which is assumed to have a predicted log depth *D* and a true log depth $D_{true}$, the scale invariance loss is defined as

(10) $$L_{si}=\frac{1}{n}\sum_{i=1}^{n}{\left(R_{i}\right)}^{2}-\frac{1}{n^{2}}{\left(\sum_{i=1}^{n}R_{i}\right)}^{2}$$

where *n* is the number of valid ground truth pixels and $R_{i}=D-D_{true}$. The multi-scale scale-invariant gradient matching loss is defined as

(11) $$L_{grad}=\frac{1}{n}\sum_{j}\sum_{i}\left(\right|\nabla_{x}R_{i}^{j}\left|+\right|\nabla_{y}R_{i}^{j}\left|\right)$$

where $\nabla_{x}$ and $\nabla_{y}$ denote the edges in the x and y directions computed by the Sobel operator, respectively, and $R_{i}^{j}$ is the logarithmic depth difference at pixel *i* at scale *j*. The total loss for each image is

(12) $$L_{tot}=L_{si}+\lambda L_{grad}$$

We set the λ=0.25.

## 4. Experiments

### 4.1. Experimental Setup

#### 4.1.1. Datasets

To verify the generalization ability of our network, we present our experimental results on two datasets, EventScape [9] and DENSE [30], which are commonly used in event and frame fusion-based monocular depth estimation research. Based on previous work [16], the EventScape dataset has a total of 734 driving data sequences, containing three subsets: the training set consisting of Town01, Town02, and Town03, and the test and validation sets from two different regions of Town05. In the DENSE dataset, the training set consists of five sequences from Town01 to Town05, respectively, the validation set consists of two sequences from Town06 and Town07, and the test set is one sequence from Town10. Each sequence consists of 1000 samples.

#### 4.1.2. Evaluation Metrics

We use absolute relative error (Abs.Rel.), log mean square error (RMSELog), scale-invariant logarithmic error (SILog), mean absolute depth error (Avg.Abs), and accuracy ($\delta <1.25^{n},n=1,2,3$) as five typical evaluation metrics, which are most widely used in depth estimation tasks. These five metrics are also evaluated at different depth thresholds (10 m, 20 m, 30 m, 80 m, 250 m, and 500 m).

#### 4.1.3. Implementation Details

We implement our network using the Pytorch framework. The basic number of channels is set to 32. During training, we use the Adam optimizer [86] for optimization and a batch size of 2. We trained our network on the EventScape dataset for 188 epochs, with the learning rate set to 0.00015 when using CAGRU-R and 0.0003 when using CAGRU-U. The learning rate was adjusted using the exponential decay method, which halves the learning rate for every 100 epochs of training. To facilitate learning, the input data are normalized, randomly cropped to a size of 224 × 224, and randomly flipped horizontally. For the voxel grid of events, their non-zero data are also normalized so that their mean and variance are 0 and 1, respectively. When training on the DENSE dataset, we first pre-train on the EventScape dataset and train the resulting model as the initial model.

### 4.2. Performance Evaluation

#### 4.2.1. Evaluation on the EventScape Dataset

**Figure 5:**
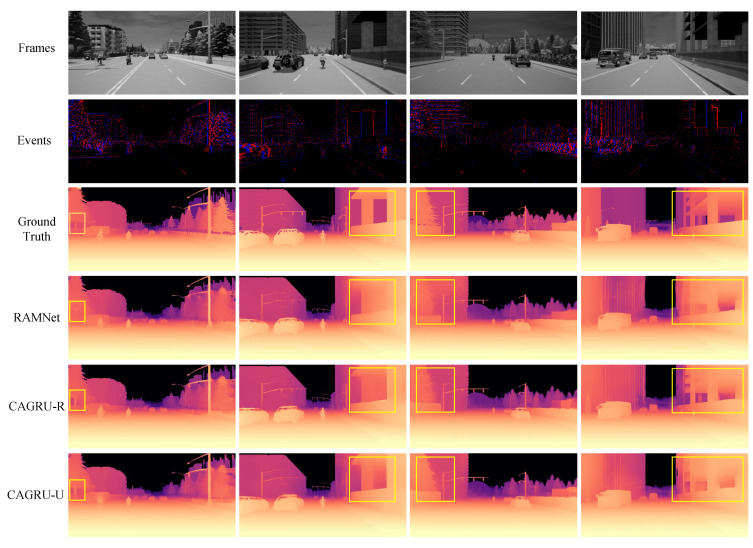
Qualitative comparison on the EventScape dataset. The samples in the first row of frame inputs are 1775, 20,681, 22,889, and 995 in order from left to right.

As shown in Table 1, we quantitatively compare our method with the state-of-the-art method RAMNet [16] on the EventScape dataset. All networks predict depth on a logarithmic scale, which is normalized and recovered to an absolute value by multiplying by the maximum depth at 80 m. The results of RAMNet are obtained by testing the pre-trained model provided in [16] on our device. Note that our method achieves the best performance among all metrics. For example, when no depth threshold is set, meaning that all pixels are considered as valid pixel points, for the absolute relative error, we obtain a gain of 0.03 and 0.033 for CAGRU-U and CAGRU-R, respectively, and our networks all achieved the best performance when the depth threshold was set. Thus, both in near- and far-range scenarios, our CAGRU is able to achieve excellent information fusion to accomplish excellent depth prediction in the network. As shown in the qualitative comparison in Figure 5, our method and RAMNet [16] do not differ much in the overall prediction results, but our method can predict clearer details in places such as tree trunks and floor edges.

Specifically, at shorter depth thresholds (e.g., 10 m, 20 m, and 30 m), our method using CAGRU-U as the state fusion operator performs the best. This indicates that, when using CAGRU-U, the model learns more fine features during training and is able to capture subtle depth differences between objects in the scene more accurately. This can also be explained by the fact that the model is able to delineate different depth levels in depth space in a more detailed way, thus providing more accurate depth information. Therefore, CAGRU-U will demonstrate its greatest advantage in some scenes that require fine depth information, such as medical image analysis and virtual reality. At larger depth thresholds (e.g., 80 m, 250 m, and 500 m), our method using CAGRU-R as the state fusion operator performs the best. This means that, when using CAGRU-R, the model pays more attention to the overall depth structure of the scene and performs better in capturing the relative depth relationships between objects in the scene, while it is less sensitive to very close or small depth differences. Therefore, CAGRU-R will be most advantageous in some scenes that require overall depth structure, such as autonomous driving and robot navigation.

#### 4.2.2. Evaluation on the DENSE Dataset

We quantitatively compare our approach on the DENSE dataset [14] with the state-of-the-art event-based methods E2Depth [14] and Spike-T [53], as well as the event and frame fusion-based methods RAMNet [16] and SRFNet [18]. All network parameters were set the same as in the EventScape dataset, where the results of our method and RAMNet are trained and tested on our device with the same settings. The data for other methods are provided by their papers. As shown in Table 2, we compare the absolute relative error (Abs.Rel), the logarithmic mean square error (RMSELog), scale-invariant logarithmic error (SILog), accuracy ($\delta <1.25^{n},n=1,2,3$), and the average absolute depth error for different depth thresholds (10 m, 20 m, and 30 m). It can be seen that our method has the best performance among all methods. For example, when the depth threshold is 10 m, for the mean absolute depth error, compared to RAMNet [16], which uses ConvGRU as the state fusion operator, we produce a gain of 0.507 using CAGRU-R as the state fusion operator and a gain of 0.485 using CAGRU-U as the state fusion operator. Since SRFNet [18] learns the more confident parts of events and frames in the form of masks, it pays more attention to the capture of edge features, so it lacks the acquisition of detailed features in the scene. E2Depth [14] and Spike-T [53] only estimate the depth from the event images, and their poor indicators further illustrate that it is difficult to obtain excellent depth prediction from sparse event images alone, and this conclusion has been confirmed in many existing works. Therefore, it is necessary to fuse the features of events and frames to predict monocular depth. Our method uses CAGRUs to make the state of event images and frame images iterate in a loop, thus achieving a perfect fusion of the two. Moreover, our method is oriented to all pixels, focusing on the more important pixels while taking into account the global features, so it performs well in detail feature extraction.

**Figure 6:**
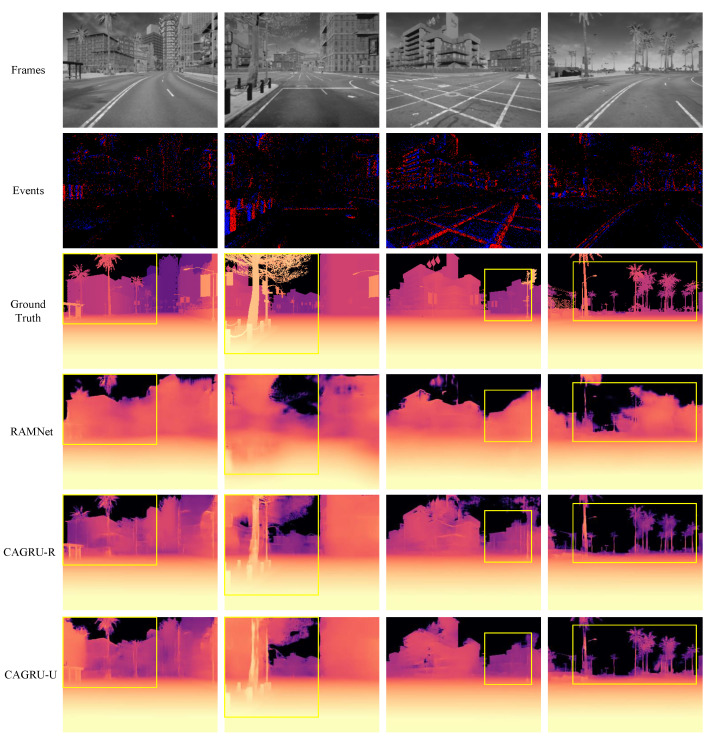
Qualitative comparison on the DENSE dataset. In the first row of frame inputs, the samples from left to right are 974, 5, 95, and 671 in that order.

We also perform a qualitative comparison between our method and RAMNet [16], as shown in Figure 6. For the samples in the first, second, and fourth columns, RAMNet [16] does not predict the trees, whereas, in the last two rows, our method predicts all the trees more clearly. As indicated in the third column of samples, RAMNet [16] predicts nearly identical depths for buildings intersecting front and back, whereas our methods all accurately predict their different depths.

### 4.3. Ablation Experiment

In addition to the performance tests, we conduct ablation experiments on the EventScape dataset to gain insight into the impact of each design choice.

#### 4.3.1. The Use of Coordinate Attention

In order to improve the performance of the model for fusing events and frame states, we add the coordinate attention gate to ConvGRU to model the temporal relationship between event sequences. The experimental setup is as follows:

- Reset gate (CAGRU-R): the coordinate attention gate is used as a reset gate, which only participates in the generation of candidate states, and the update gate adopts a convolutional gate;
- Update gate (CAGRU-U): the coordinate attention gate is used as an update gate, which only participates in the update of the hidden state, and the reset gate adopts a convolutional gate;
- Reset gate + Update gate: the coordinate attention gate is used as both a reset gate and an update gate, and no convolutional gates are used in the whole unit;
- State: the previous state and the current feature are re-weighted by the coordinate attention gate and then participate in the generation of candidate states as the previous state again, and the convolutional gate is still used for both the reset gate and the update gate.

As shown in Table 3, we compare absolute relative error, squared relative error, and mean absolute depth error at 10 m, 20 m, and 30 m depth thresholds. “Reset gate (CAGRU-R)” provides the best performance in all metrics, and “Update gate (CAGRU-U)” is only second to it. Both methods are implemented cooperatively by a coordinate attention gate and a convolutional gate and have achieved excellent results in feature extraction. The poor performance of ‘Reset gate + Update gate’ may be due to the fact that the coordinate attention gate enhances the channel coding capability of the model, but it also needs to be combined with the spatial encoding capability of the convolution. A combination of the coordinate attention gate and the convolution gate is the best choice. “State” has the worst effect because the previous state has been screened for retainable states under the coordinate attention gate, but the subsequent update gate screens it again, making useful information somewhat lost.

#### 4.3.2. Selection of Channel Reduction Factors

In order to investigate the effect of different channel reduction factors (r in Figure 3) in the coordinate attention, we try to reduce the size of the reduction factor and observe the change in performance. As shown in Table 4, we find that, as the reduction factor decreases, the number of parameters increases, but there is no significant difference in model performance. It can be seen that the performance of our model depends very little on the reduction factor, and, regardless of whether the reduction factor is increased or decreased, our model can maintain good performance while reducing the amount of computation.

## 5. Conclusions

In this work, we introduce the CAGRU, a new gated recurrent unit, which is used for state fusion of data with different modalities. It adopts coordinate attention as a coordinate attention gate to model inter-channel relationships. We embed the CAGRU into a two-stream encoding–decoding network structure for monocular depth estimation. The experiments on the EventScape and DENSE datasets show that our CAGRU outperforms the ConvGRU used in the state-of-the-art method RAMNet. This is because our CAGRU retains a convolutional gate while introducing the coordinate attention gate, which makes the two cooperate with each other to model comprehensive information in time, space, and channels. When fusing information from different modalities, a CAGRU can selectively extract more important information, which lays a solid foundation for the network to achieve excellent monocular depth estimation.

Our model takes only 0.04 s to predict the depth information when processing a sequence containing six images. As a result, our model has low latency in predicting depth information, thereby effectively improving the responsiveness and safety of autonomous vehicles.

By employing two large and widely used synthetic datasets to train the model, we fully utilize the significant advantages of synthetic data in providing rich and diverse samples and overcoming the scarcity of real data. These synthetic datasets not only helped us to effectively explore the performance boundaries of the models but also facilitated the algorithm’s generalization ability in complex scenarios. However, there are some limitations regarding synthetic datasets, mainly including the imperfect match with the real-world data distribution, which may lead to deviations in model performance in real applications, as well as the potential bias and noise that may be introduced during the synthesis process, which need to be overcome by more refined data generation techniques and domain generalization strategies in future research.

## Figures and Tables

**Table 1:** Quantitative results on the EventScape dataset. All methods were trained on EventScape’s Town01–Town03 and validated and tested on Town05.

Depth Threshold	Method	Abs.Rel↓	RMSELog↓	SILog↓	Avg.Abs↓	δ<1.25↑	$\delta <1.25^{2}$↑	$\delta <1.25^{3}$↑
None	RAMNet	0.198	0.355	0.116	13.878	0.79	0.891	0.946
	CAGRU-R	**0.165**	**0.298**	**0.075**	**10.924**	**0.819**	**0.916**	**0.961**
	CAGRU-U	0.168	0.168	0.168	0.168	0.168	0.168	0.168
10 m	RAMNet	0.109	0.128	0.024	0.609	0.955	0.977	0.988
	CAGRU-R	0.091	0.112	0.022	0.502	0.963	0.982	0.99
	CAGRU-U	**0.08**	**0.099**	**0.018**	**0.434**	**0.969**	**0.987**	**0.993**
20 m	RAMNet	0.152	0.192	0.038	1.478	0.904	0.956	0.982
	CAGRU-R	0.128	0.174	0.033	1.233	0.918	0.966	0.985
	CAGRU-U	**0.114**	**0.162**	**0.029**	**1.121**	**0.925**	**0.971**	**0.987**
30 m	RAMNet	0.185	0.232	0.049	2.528	0.864	0.938	0.977
	CAGRU-R	0.154	0.209	0.042	2.105	0.885	0.952	0.981
	CAGRU-U	**0.144**	**0.205**	**0.04**	**2.049**	**0.887**	**0.953**	**0.981**
80 m	RAMNet	0.238	0.309	0.074	6.421	0.774	0.892	0.953
	CAGRU-R	**0.198**	**0.269**	**0.058**	**5.345**	**0.806**	**0.916**	**0.967**
	CAGRU-U	0.2	0.287	0.066	5.776	0.797	0.903	0.956
250 m	RAMNet	0.246	0.331	0.082	8.594	0.751	0.876	0.944
	CAGRU-R	**0.206**	**0.287**	**0.065**	**7.22**	**0.784**	**0.904**	**0.96**
	CAGRU-U	0.21	0.311	0.075	7.933	0.773	0.888	0.946
500 m	RAMNet	0.248	0.339	0.086	9.873	0.746	0.871	0.94
	CAGRU-R	**0.208**	**0.298**	**0.069**	**8.476**	**0.779**	**0.9**	**0.956**
	CAGRU-U	0.211	0.318	0.078	9.005	0.769	0.884	0.944

↓ indicates lower is better; ↑ indicates higher is better. Bolded is best, and underlined is second. The depth threshold represents the depth boundaries in the scene and is used to select valid depth data. When the predicted depth value of an object in the scene is less than the depth threshold, it is considered as foreground; when its predicted depth is greater than the depth threshold, it is considered as background.

**Table 2:** Performance evaluation on the DENSE dataset. / indicates that these data are not provided in the paper; bolded is best, and underlined is second best.

Method	Input	Abs.Rel↓	RMSELog↓	SILog↓	δ<1.25↑	$\delta <1.25^{2}$↑	$\delta <1.25^{3}$↑	10 m↓	20 m↓	30 m↓
E2Depth [14]	E	0.674	0.765	0.441	0.639	0.729	0.789	/	/	/
Spike-T [53]	E	0.606	0.706	0.395	0.682	0.762	0.813	/	/	/
RAMNet [16]	E+I	0.743	0.849	0.602	0.678	0.761	0.818	1.111	6.806	12.878
SRFNet [18]	E+I	0.513	0.687	/	/	/	/	1.503	3.566	6.116
CAGRU-R	E+I	0.35	**0.537**	0.277	**0.735**	**0.83**	**0.881**	**0.604**	2.305	4.579
CAGRU-U	E+I	**0.307**	0.558	**0.225**	0.725	0.815	0.869	0.626	**2.139**	**2.823**

**Table 3:** Depth estimation performance for different usages of coordinate attention in ConvGRU. Bolded is best, and underlined is second.

Method	Abs.Rel↓	Sq.Rel↓	10 m↓	20 m↓	30 m↓
Reset gate(CAGRU-R)	**0.182**	**2.472**	**0.516**	**1.305**	**2.353**
Update gate(CAGRU-U)	0.184	3.029	0.586	1.392	2.368
Reset gate + Update gate	0.187	3.198	0.569	1.409	2.43
State	0.208	3.624	0.658	1.785	2.882

**Table 4:** Comparison of monocular depth estimation performance with different attentional reduction factors. Bolded is best, and underlined is second.

Reduction	Parameter	Abs.Rel↓	RMSELog↓	10 m↓	20 m↓	30 m↓
32	12.18M	**0.182**	0.319	**0.516**	**1.305**	**2.353**
16	12.22M	0.226	0.322	0.545	1.834	3.208
8	12.31M	0.184	**0.316**	0.536	1.406	2.462

## Data Availability

The raw data supporting the conclusions of this article will be made available by the authors on request.
